# (±)-*trans*-6,7-Dimeth­oxy-1-oxo-3-(2-thien­yl)isochroman-4-carboxylic acid

**DOI:** 10.1107/S1600536809018844

**Published:** 2009-05-23

**Authors:** Mehmet Akkurt, Zeliha Baktır, Milen G. Bogdanov, Ivan V. Svinyarov, Orhan Büyükgüngör

**Affiliations:** a Department of Physics, Faculty of Arts and Sciences, Erciyes University, 38039 Kayseri, Turkey; b Faculty of Chemistry, University of Sofia, 1 James Bourchier Boulevard, 1164 Sofia, Bulgaria; c Department of Physics, Faculty of Arts and Sciences, Ondokuz Mayıs University, 55139 Samsun, Turkey

## Abstract

The title compound, C_16_H_14_O_6_S, was synthesized by the reaction of 6,7-dimethoxy­homophthalic anhydride with thio­phene-2-carbaldehyde in the presence of 4-(dimethyl­amino)pyridine (DMAP) as a basic catalyst. The thio­phene ring of the title mol­ecule is disordered over two sites with occupancies of 0.877 (3) and 0.123 (3). The disorder corresponds to an approximate 180° rotation of the thio­phene ring with respect to the C—C bond linking it to the rest of the mol­ecule. The six-membered ring of the 3,4-dihydro­isochromanone ring system is not planar [puckering parameters *Q*_T_ = 0.571 (2) Å, θ = 115.2 (2)° and ϕ = 99.1 (2)°]. The benzene ring of the 3,4-dihydro­isochromanone ring system makes dihedral angles of 75.0 (2) and 77.2 (5)° with the disordered thio­phene rings. Inter­molecular O—H⋯O and C—H⋯O hydrogen bonds, as well as C—H⋯π inter­actions, lead to the observed supra­molecular structure.

## Related literature

For details of the synthesis of the title compound, see: Bogdanov & Palamareva (2004 ▶). For the synthesis of new dihydro­isocoumarins, see: Bogdanov *et al.* (2007*a* ▶,*b* ▶). For ring-puckering parameters, see: Cremer & Pople (1975 ▶).
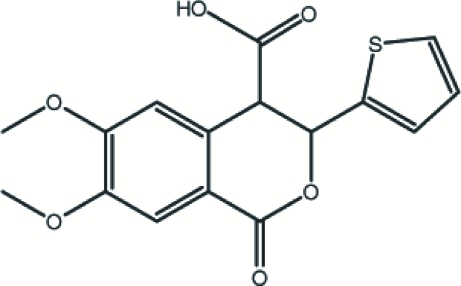


## Experimental

### 

#### Crystal data

C_16_H_14_O_6_S
*M* *_r_* = 334.34
Triclinic, 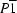

*a* = 8.3369 (6) Å
*b* = 8.4587 (6) Å
*c* = 11.9143 (9) Å
α = 76.441 (6)°
β = 81.127 (6)°
γ = 72.958 (6)°
*V* = 777.6 (1) Å^3^
*Z* = 2
Mo *K*α radiation
μ = 0.24 mm^−1^
*T* = 296 K
0.53 × 0.41 × 0.21 mm

#### Data collection

Stoe IPDS II diffractometer
Absorption correction: integration (*X-RED32*; Stoe & Cie, 2002 ▶) *T*_min_ = 0.885, *T*_max_ = 0.952
8329 measured reflections
3229 independent reflections
2663 reflections with *I* > 2σ(*I*)
*R*_int_ = 0.030

#### Refinement

*R*[*F* ^2^ > 2σ(*F* ^2^)] = 0.044
*wR*(*F* ^2^) = 0.113
*S* = 1.07
3229 reflections
219 parameters
13 restraints
H atoms treated by a mixture of independent and constrained refinement
Δρ_max_ = 0.27 e Å^−3^
Δρ_min_ = −0.31 e Å^−3^

### 

Data collection: *X-AREA* (Stoe & Cie, 2002 ▶); cell refinement: *X-AREA*; data reduction: *X-RED32* (Stoe & Cie, 2002 ▶); program(s) used to solve structure: *SIR97* (Altomare *et al.*, 1999 ▶); program(s) used to refine structure: *SHELXL97* (Sheldrick, 2008 ▶); molecular graphics: *ORTEP-3 for Windows* (Farrugia, 1997 ▶); software used to prepare material for publication: *WinGX* (Farrugia, 1999 ▶).

## Supplementary Material

Crystal structure: contains datablocks global, I. DOI: 10.1107/S1600536809018844/im2117sup1.cif

Structure factors: contains datablocks I. DOI: 10.1107/S1600536809018844/im2117Isup2.hkl

Additional supplementary materials:  crystallographic information; 3D view; checkCIF report

## Figures and Tables

**Table 1:** Hydrogen-bond geometry (Å, °)

*D*—H⋯*A*	*D*—H	H⋯*A*	*D*⋯*A*	*D*—H⋯*A*
O6—H6⋯O3^i^	0.86 (3)	1.84 (2)	2.658 (2)	159 (3)
C2—H2⋯O5^ii^	0.93	2.54	3.465 (2)	172
C10—H10⋯O6^iii^	0.98	2.54	3.475 (2)	159
C11—H11⋯*Cg*4^iv^	0.98	2.61	3.525 (2)	156

Symmetry codes: (i) 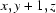; (ii) 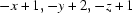; (iii) 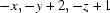; (iv) 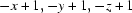. *Cg*4 is the centroid of the C1–C6 ring.

**Fig. 1.**
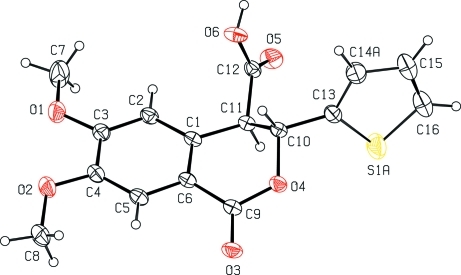
The title molecule of (I) with the atom-labelling scheme and displacement ellipsoids drawn at the 30% probability level. The minor component of the disorder has been omitted.

**Fig. 2.**
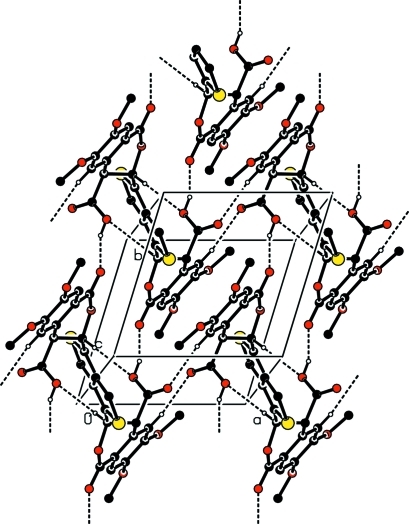
Perspective view of the intermolecular hdrogen bonding interactions in the structure of (I). H atoms not involved in hydrogen bonding and the minor component of the disordered thiophene ring have been omitted for clarity.

## supplementary crystallographic information

### Comment

The title compound (I) was obtained as a part of a research project aimed at the synthesis of new dihydroisocoumarins with potential pharmacological activities (Bogdanov *et al.*, 2007*a*,*b*). (I) was synthesized by a one-pot reaction of 6,7-dimethoxyhomophthalic anhydride with thiophene-2-carboxaldehyde in the presence of DMAP as a basic catalyst (Bogdanov & Palamareva, 2004). The structure of (I) was determined by spectral methods (^1^H NMR & IR) and microanalysis. In this paper, we report the X-ray crystallographic study of (I).

In the title molecule, (I), the thiophene ring is disordered over two sites and the major component of the disorder labelled with suffix A is shown in Fig. 1. The disorder corresponds to an approximate 180° rotation with respect to the C10—C13 bond. The six-membered ring (O4/C1/C6/C9–C11) of the 3,4-dihydroisochromanone ring system is not planar, showing the puckering parameters: Q_T_ = 0.571 (2) Å, θ = 115.2 (2)° and φ = 99.1 (2)° (Cremer & Pople, 1975). The benzene ring (C1–C6) of the 3,4-dihydroisochromanone ring system encloses dihedral angles of 75.0 (2)° and 77.2 (5)° with the thiophene rings A(C13/C14A/C15/C16/S1A) and B (C13/C14B/C15/C16/S1B), respectively.

The crystal structure is realized by intermolecular O—H···O and C—H···O hydrogen bonds and C—H···π interactions (Table 1, Fig. 2).

### Experimental

Compound (I) was synthesized by the reaction of 6,7-dimethoxyhomophthalic anhydride (1) with thiophene-2-carbaldehyde (2) in the presence of DMAP as a basic catalyst (Bogdanov & Palamareva, 2004). To a mixture of 1 (0.33 g, 1.5 mmol) and 2 (0.15 ml, 1.65 mmol) in dry chloroform (5 ml) DMAP (0.18 g, 1.5 mmol) was added. The mixture was stirred at room temperature for 1 h. At the end of the reaction (monitored by TLC), the reaction mixture was extracted with 10% sodium hydrogen carbonate. The aqueous layer was further acidified (pH = 3) with 10% hydrochloric acid and extracted with ethyl acetate. The organic layer was dried (sodium sulfate), filtered and the solvent was then evaporated under reduced pressure giving (0.49 g, 98%) of a diastereomeric mixture of *cis*- and *trans*-(±)-6,7-dimethoxy-1-oxo-3-(thiophen-2-yl)isochroman-4-carboxylic acids, in a 30:70 ratio, favoring the *trans* diastereomer. Pure *trans*-diastereomer (I) was obtained by fractional crystallization of the residue from ethyl acetate. The product was characterized by ^1^H NMR, IR spectra and elemental analysis. Single crystals were obtained by slow evaporation of a chloroform–ethyl acetate (3:1) solution of (I) at room temperature (m.p. 465–467 K). Analysis, calculated for C_16_H_14_O_6_S (334.34): C 57.48, H 4.22, O 28.71, S 9.59 (%); found: C 57.74, H 3.97, O 28.71, S 9.91 (%). IR (KBr) 1693 cm^-1^ (C═O), 1741 cm^-1^ (C═O). The ^1^H NMR spectrum of (I) was obtained on a Bruker DRX-250 spectrometer at 250.13 MHz. Chemical shifts (δ) are expressed in parts per million (p.p.m.). ^1^H NMR (250 MHz, DMSO-d_6_) δ = 3.82 (3H, s, –O—CH_3_), 3.85 (3H, s, –O—CH_3_), 4.43 (1H, d, J = 3 Hz, H-4), 6.29 (1H, d, J = 3 Hz, H-3), 6.96 (1H, dd, J = 3.6 and 5 Hz, Th—H), 7.08 (1H, s, Ph—H), 7.13 (1H, d, J = 3.5 Hz, Th—H), 7.37 (1H, s, Ph—H), 7.44 (1H, dd, J = 1 and 5 Hz, Th—H).

### Refinement

The H atom of the hydroxyl group was found from a difference Fourier map and refined freely [O6—H6 = 0.864 (3) Å]. H atoms bonded to C atoms were placed at calculated positions with the C—H distances in a range of 0.93–0.98 Å, and were included in the refinement in the riding-model approximation, with *U*_iso_(H) = 1.2 or 1.5*U*_eq_(C). The ratio of the refined site occupancies for the major and minor components of the disordered thiophene ring is 0.877 (3):0.123 (3). Similarity restraints were applied to the displacement parameters of the disordered atoms, and there were also geometrical restraints.

### Crystal data

**Table:**

C_16_H_14_O_6_S	*Z* = 2
*M**_r_* = 334.34	*F*(000) = 348
Triclinic, *P*1	*D*_x_ = 1.428 Mg m^−^^3^
Hall symbol: -P 1	Mo *K*α radiation, λ = 0.71073 Å
*a* = 8.3369 (6) Å	Cell parameters from 16761 reflections
*b* = 8.4587 (6) Å	θ = 2.6–28.0°
*c* = 11.9143 (9) Å	µ = 0.24 mm^−^^1^
α = 76.441 (6)°	*T* = 296 K
β = 81.127 (6)°	Prism, colourless
γ = 72.958 (6)°	0.53 × 0.41 × 0.21 mm
*V* = 777.6 (1) Å^3^	

### Data collection

**Table:**

Stoe IPDS II diffractometer	3229 independent reflections
Radiation source: sealed X-ray tube, 12 x 0.4 mm long-fine focus	2663 reflections with *I* > 2σ(*I*)
plane graphite	*R*_int_ = 0.030
Detector resolution: 6.67 pixels mm^-1^	θ_max_ = 26.5°, θ_min_ = 2.6°
ω scans	*h* = −10→10
Absorption correction: integration (*X-RED32*; Stoe & Cie, 2002)	*k* = −10→10
*T*_min_ = 0.885, *T*_max_ = 0.952	*l* = −14→14
8329 measured reflections	

### Refinement

**Table:**

Refinement on *F*^2^	Primary atom site location: structure-invariant direct methods
Least-squares matrix: full	Secondary atom site location: difference Fourier map
*R*[*F*^2^ > 2σ(*F*^2^)] = 0.044	Hydrogen site location: inferred from neighbouring sites
*wR*(*F*^2^) = 0.113	H atoms treated by a mixture of independent and constrained refinement
*S* = 1.07	*w* = 1/[σ^2^(*F*_o_^2^) + (0.0514*P*)^2^ + 0.2422*P*] where *P* = (*F*_o_^2^ + 2*F*_c_^2^)/3
3229 reflections	(Δ/σ)_max_ < 0.001
219 parameters	Δρ_max_ = 0.27 e Å^−^^3^
13 restraints	Δρ_min_ = −0.31 e Å^−^^3^

### Special details

**Table:**

Geometry. Bond distances, angles *etc*. have been calculated using the rounded fractional coordinates. All su's are estimated from the variances of the (full) variance-covariance matrix. The cell e.s.d.'s are taken into account in the estimation of distances, angles and torsion angles
Refinement. Refinement on *F*^2^ for ALL reflections except those flagged by the user for potential systematic errors. Weighted *R*-factors *wR* and all goodnesses of fit *S* are based on *F*^2^, conventional *R*-factors *R* are based on *F*, with *F* set to zero for negative *F*^2^. The observed criterion of *F*^2^ > σ(*F*^2^) is used only for calculating -*R*-factor-obs *etc*. and is not relevant to the choice of reflections for refinement. *R*-factors based on *F*^2^ are statistically about twice as large as those based on *F*, and *R*-factors based on ALL data will be even larger.

### Fractional atomic coordinates and isotropic or equivalent isotropic displacement parameters (Å^2^)

**Table:**

	*x*	*y*	*z*	*U*_iso_*/*U*_eq_	Occ. (<1)
S1A	0.17140 (13)	0.63559 (11)	0.85712 (7)	0.0759 (3)	0.877 (3)
O1	0.5188 (2)	0.77377 (19)	0.14713 (12)	0.0557 (5)	
O2	0.38518 (19)	0.54308 (18)	0.12791 (12)	0.0503 (5)	
O3	0.15691 (17)	0.32562 (15)	0.54690 (12)	0.0445 (4)	
O4	0.13106 (15)	0.53400 (15)	0.63275 (10)	0.0370 (4)	
O5	0.42866 (19)	0.91589 (18)	0.62175 (13)	0.0515 (5)	
O6	0.22136 (17)	1.01715 (16)	0.50460 (13)	0.0451 (4)	
C1	0.33543 (19)	0.67769 (19)	0.44799 (14)	0.0304 (4)	
C2	0.4244 (2)	0.7526 (2)	0.35183 (16)	0.0356 (5)	
C3	0.4377 (2)	0.7065 (2)	0.24602 (16)	0.0384 (5)	
C4	0.3637 (2)	0.5802 (2)	0.23513 (15)	0.0372 (5)	
C5	0.2797 (2)	0.5039 (2)	0.33076 (16)	0.0359 (5)	
C6	0.26476 (19)	0.55214 (19)	0.43731 (15)	0.0311 (5)	
C7	0.6107 (4)	0.8880 (4)	0.1547 (2)	0.0869 (13)	
C8	0.3476 (3)	0.3914 (3)	0.12059 (19)	0.0611 (8)	
C9	0.1816 (2)	0.4620 (2)	0.53946 (15)	0.0330 (5)	
C10	0.1347 (2)	0.7092 (2)	0.61942 (15)	0.0319 (5)	
C11	0.31183 (19)	0.72133 (19)	0.56665 (14)	0.0305 (4)	
C12	0.3300 (2)	0.8940 (2)	0.56779 (15)	0.0339 (5)	
C13	0.0822 (2)	0.7555 (2)	0.73510 (16)	0.0385 (5)	
C14A	−0.0344 (8)	0.8941 (8)	0.7626 (6)	0.0585 (17)	0.877 (3)
C15	−0.0506 (4)	0.9063 (4)	0.8781 (2)	0.0802 (8)	
C16	0.0534 (4)	0.7747 (4)	0.9384 (2)	0.0802 (8)	
S1B	−0.0621 (16)	0.9313 (15)	0.7433 (10)	0.058 (3)	0.123 (3)
C14B	0.144 (3)	0.692 (3)	0.8422 (15)	0.0802 (8)	0.123 (3)
H5	0.23240	0.41970	0.32480	0.0430*	
H6	0.227 (3)	1.112 (3)	0.516 (2)	0.064 (7)*	
H7A	0.69670	0.83190	0.20660	0.1050*	
H7B	0.53550	0.98290	0.18340	0.1050*	
H7C	0.66180	0.92640	0.07920	0.1050*	
H8A	0.23190	0.39800	0.14800	0.0730*	
H2	0.47510	0.83390	0.35870	0.0430*	
H8C	0.36690	0.37790	0.04140	0.0730*	
H10	0.05270	0.78130	0.56540	0.0380*	
H11	0.39450	0.63610	0.61480	0.0370*	
H14A	−0.09870	0.97510	0.70800	0.0700*	0.877 (3)
H15	−0.12440	0.99480	0.90910	0.0960*	
H16	0.06060	0.76040	1.01750	0.0960*	
H8B	0.41900	0.29650	0.16740	0.0730*	
H14B	0.23880	0.60080	0.85330	0.0960*	0.123 (3)

### Atomic displacement parameters (Å^2^)

**Table:**

	*U*^11^	*U*^22^	*U*^33^	*U*^12^	*U*^13^	*U*^23^
S1A	0.0947 (6)	0.0739 (6)	0.0417 (4)	0.0110 (4)	−0.0144 (3)	−0.0153 (3)
O1	0.0752 (10)	0.0589 (9)	0.0414 (8)	−0.0358 (8)	0.0138 (7)	−0.0161 (6)
O2	0.0680 (9)	0.0552 (8)	0.0366 (7)	−0.0240 (7)	−0.0013 (6)	−0.0192 (6)
O3	0.0519 (7)	0.0311 (6)	0.0557 (8)	−0.0171 (6)	0.0018 (6)	−0.0156 (6)
O4	0.0425 (6)	0.0332 (6)	0.0399 (7)	−0.0162 (5)	0.0033 (5)	−0.0132 (5)
O5	0.0596 (8)	0.0501 (8)	0.0586 (9)	−0.0260 (7)	−0.0186 (7)	−0.0145 (7)
O6	0.0488 (7)	0.0274 (6)	0.0646 (9)	−0.0079 (5)	−0.0164 (6)	−0.0154 (6)
C1	0.0262 (7)	0.0273 (7)	0.0391 (9)	−0.0044 (6)	−0.0033 (6)	−0.0128 (6)
C2	0.0346 (8)	0.0336 (8)	0.0430 (9)	−0.0126 (7)	0.0006 (7)	−0.0144 (7)
C3	0.0391 (9)	0.0362 (9)	0.0392 (9)	−0.0089 (7)	0.0020 (7)	−0.0112 (7)
C4	0.0379 (9)	0.0384 (9)	0.0370 (9)	−0.0062 (7)	−0.0049 (7)	−0.0152 (7)
C5	0.0342 (8)	0.0349 (8)	0.0437 (10)	−0.0099 (7)	−0.0054 (7)	−0.0157 (7)
C6	0.0264 (7)	0.0287 (8)	0.0395 (9)	−0.0046 (6)	−0.0035 (6)	−0.0128 (7)
C7	0.129 (3)	0.100 (2)	0.0588 (15)	−0.084 (2)	0.0344 (16)	−0.0281 (15)
C8	0.0914 (17)	0.0613 (13)	0.0447 (12)	−0.0308 (13)	−0.0033 (11)	−0.0266 (10)
C9	0.0298 (8)	0.0292 (8)	0.0422 (9)	−0.0064 (6)	−0.0041 (7)	−0.0131 (7)
C10	0.0324 (8)	0.0282 (8)	0.0373 (9)	−0.0086 (6)	−0.0015 (7)	−0.0113 (6)
C11	0.0296 (7)	0.0270 (7)	0.0365 (9)	−0.0060 (6)	−0.0041 (6)	−0.0108 (6)
C12	0.0349 (8)	0.0357 (8)	0.0364 (9)	−0.0137 (7)	0.0009 (7)	−0.0150 (7)
C13	0.0384 (9)	0.0389 (9)	0.0404 (10)	−0.0109 (7)	0.0015 (7)	−0.0148 (7)
C14A	0.066 (3)	0.053 (3)	0.047 (3)	0.001 (2)	−0.0090 (19)	−0.011 (2)
C15	0.1059 (16)	0.0819 (14)	0.0539 (11)	−0.0163 (11)	0.0061 (10)	−0.0355 (10)
C16	0.1059 (16)	0.0819 (14)	0.0539 (11)	−0.0163 (11)	0.0061 (10)	−0.0355 (10)
S1B	0.065 (4)	0.054 (5)	0.041 (4)	0.015 (3)	−0.008 (3)	−0.020 (4)
C14B	0.1059 (16)	0.0819 (14)	0.0539 (11)	−0.0163 (11)	0.0061 (10)	−0.0355 (10)

### Geometric parameters (Å, °)

**Table:**

S1A—C13	1.702 (2)	C10—C13	1.487 (2)
S1A—C16	1.689 (3)	C10—C11	1.536 (2)
S1B—C15	1.584 (12)	C11—C12	1.515 (2)
S1B—C13	1.628 (13)	C13—C14B	1.379 (19)
O1—C7	1.423 (4)	C13—C14A	1.357 (7)
O1—C3	1.354 (2)	C14A—C15	1.387 (7)
O2—C8	1.431 (3)	C14B—C16	1.47 (2)
O2—C4	1.359 (2)	C15—C16	1.326 (4)
O3—C9	1.211 (2)	C2—H2	0.9300
O4—C9	1.344 (2)	C5—H5	0.9300
O4—C10	1.462 (2)	C7—H7A	0.9600
O5—C12	1.197 (2)	C7—H7B	0.9600
O6—C12	1.327 (2)	C7—H7C	0.9600
O6—H6	0.86 (3)	C8—H8A	0.9600
C1—C11	1.513 (2)	C8—H8B	0.9600
C1—C2	1.392 (2)	C8—H8C	0.9600
C1—C6	1.394 (2)	C10—H10	0.9800
C2—C3	1.384 (3)	C11—H11	0.9800
C3—C4	1.419 (2)	C14A—H14A	0.9300
C4—C5	1.371 (3)	C14B—H14B	0.9300
C5—C6	1.399 (2)	C15—H15	0.9300
C6—C9	1.469 (2)	C16—H16	0.9300
			
S1A···O4	3.0857 (15)	C3···H11^viii^	3.0300
S1A···C8^i^	3.589 (2)	C4···H11^viii^	2.8300
S1B···O6^ii^	3.308 (12)	C5···H8B	2.8100
S1B···O6	3.488 (13)	C5···H11^viii^	2.7400
S1B···C12	3.571 (13)	C5···H8A	2.6600
S1B···C2^ii^	3.530 (13)	C6···H10	2.7900
S1B···C3^ii^	3.694 (13)	C6···H11^viii^	2.8700
S1A···H8C^i^	3.0000	C7···H2	2.5200
S1A···H11	3.1800	C8···H5	2.5100
O1···O2	2.581 (2)	C9···H11	2.9600
O2···O1	2.581 (2)	C9···H6^iii^	2.95 (2)
O3···O6^iii^	2.6575 (18)	C12···H2	2.6900
O3···C6^iv^	3.350 (2)	H2···O6	2.8500
O3···O4^iv^	3.2369 (19)	H2···C7	2.5200
O3···C9^iv^	3.051 (2)	H2···C12	2.6900
O4···O3^iv^	3.2369 (19)	H2···H7A	2.3800
O4···S1A	3.0857 (15)	H2···H7B	2.2400
O5···O6^v^	3.212 (2)	H2···O5^v^	2.5400
O5···O5^v^	3.138 (2)	H5···O3	2.6100
O5···C12^v^	3.244 (2)	H5···C8	2.5100
O5···C7^v^	3.382 (3)	H5···H8A	2.1600
O6···S1B^ii^	3.308 (12)	H5···H8B	2.4700
O6···C2	3.150 (2)	H5···O4^iv^	2.9200
O6···O3^vi^	2.6575 (18)	H6···O3^vi^	1.84 (2)
O6···S1B	3.488 (13)	H6···C9^vi^	2.95 (2)
O6···C13	3.385 (2)	H6···H10^ii^	2.5000
O6···O5^v^	3.212 (2)	H7A···C2	2.7800
O1···H8C^vii^	2.7500	H7A···H2	2.3800
O2···H8C^vii^	2.7700	H7B···C2	2.7100
O3···H6^iii^	1.84 (2)	H7B···H2	2.2400
O3···H10^iv^	2.8100	H7B···H8B^vi^	2.5200
O3···H5	2.6100	H7B···O5^v^	2.7400
O4···H5^iv^	2.9200	H7C···H15^x^	2.5600
O5···H2^v^	2.5400	H8A···C5	2.6600
O5···H7B^v^	2.7400	H8A···H5	2.1600
O6···H14A^ii^	2.8500	H8B···C5	2.8100
O6···H10	2.6700	H8B···H5	2.4700
O6···H2	2.8500	H8B···H7B^iii^	2.5200
O6···H10^ii^	2.5400	H8C···S1A^ix^	3.0000
C1···C1^viii^	3.545 (2)	H8C···O1^vii^	2.7500
C2···S1B^ii^	3.530 (13)	H8C···O2^vii^	2.7700
C2···C9^viii^	3.532 (2)	H10···O6	2.6700
C2···O6	3.150 (2)	H10···C6	2.7900
C3···S1B^ii^	3.694 (13)	H10···H14A	2.5700
C6···O3^iv^	3.350 (2)	H10···O3^iv^	2.8100
C7···O5^v^	3.382 (3)	H10···O6^ii^	2.5400
C8···S1A^ix^	3.589 (2)	H10···H6^ii^	2.5000
C9···C9^iv^	3.150 (2)	H11···S1A	3.1800
C9···C2^viii^	3.532 (2)	H11···C9	2.9600
C9···O3^iv^	3.051 (2)	H11···C1^viii^	3.1000
C12···O5^v^	3.244 (2)	H11···C3^viii^	3.0300
C12···S1B	3.571 (13)	H11···C4^viii^	2.8300
C12···C14A	3.527 (7)	H11···C5^viii^	2.7400
C13···O6	3.385 (2)	H11···C6^viii^	2.8700
C14A···C12	3.527 (7)	H14A···H10	2.5700
C1···H11^viii^	3.1000	H14A···O6^ii^	2.8500
C2···H7B	2.7100	H14A···C2^ii^	3.0600
C2···H14A^ii^	3.0600	H15···H7C^xi^	2.5600
C2···H7A	2.7800		
			
C13—S1A—C16	91.85 (12)	C13—C14A—C15	115.2 (5)
C13—S1B—C15	92.3 (7)	C13—C14B—C16	117.2 (17)
C3—O1—C7	117.71 (16)	C14A—C15—C16	110.8 (4)
C4—O2—C8	116.56 (15)	S1B—C15—C16	123.5 (5)
C9—O4—C10	117.75 (13)	S1A—C16—C15	113.17 (19)
C12—O6—H6	108.4 (17)	C14B—C16—C15	98.8 (9)
C6—C1—C11	116.64 (14)	C1—C2—H2	120.00
C2—C1—C6	119.26 (15)	C3—C2—H2	120.00
C2—C1—C11	124.09 (15)	C4—C5—H5	120.00
C1—C2—C3	120.20 (16)	C6—C5—H5	120.00
O1—C3—C2	124.71 (16)	O1—C7—H7A	110.00
C2—C3—C4	120.36 (16)	O1—C7—H7B	109.00
O1—C3—C4	114.93 (16)	O1—C7—H7C	109.00
C3—C4—C5	119.21 (16)	H7A—C7—H7B	109.00
O2—C4—C5	125.01 (16)	H7A—C7—H7C	109.00
O2—C4—C3	115.78 (16)	H7B—C7—H7C	109.00
C4—C5—C6	120.31 (16)	O2—C8—H8A	109.00
C5—C6—C9	118.97 (15)	O2—C8—H8B	109.00
C1—C6—C5	120.64 (16)	O2—C8—H8C	109.00
C1—C6—C9	120.30 (15)	H8A—C8—H8B	109.00
O4—C9—C6	118.04 (14)	H8A—C8—H8C	109.00
O3—C9—O4	117.07 (16)	H8B—C8—H8C	109.00
O3—C9—C6	124.89 (16)	O4—C10—H10	109.00
O4—C10—C13	107.21 (13)	C11—C10—H10	109.00
O4—C10—C11	107.57 (13)	C13—C10—H10	109.00
C11—C10—C13	115.54 (14)	C1—C11—H11	108.00
C1—C11—C10	106.68 (13)	C10—C11—H11	108.00
C10—C11—C12	109.46 (13)	C12—C11—H11	108.00
C1—C11—C12	115.26 (14)	C13—C14A—H14A	122.00
O6—C12—C11	112.16 (15)	C15—C14A—H14A	122.00
O5—C12—C11	123.52 (16)	C16—C14B—H14B	121.00
O5—C12—O6	124.30 (16)	C13—C14B—H14B	121.00
C10—C13—C14A	128.5 (3)	C16—C15—H15	125.00
S1A—C13—C10	122.57 (13)	S1B—C15—H15	112.00
S1A—C13—C14A	109.0 (3)	C14A—C15—H15	125.00
S1B—C13—C14B	107.5 (11)	C14B—C16—H16	138.00
S1B—C13—C10	118.1 (4)	S1A—C16—H16	123.00
C10—C13—C14B	133.8 (10)	C15—C16—H16	123.00
			
C16—S1A—C13—C14A	−0.8 (4)	O1—C3—C4—C5	179.60 (16)
C13—S1A—C16—C15	0.5 (3)	O2—C4—C5—C6	−179.91 (16)
C16—S1A—C13—C10	177.53 (18)	C3—C4—C5—C6	1.1 (3)
C7—O1—C3—C2	6.4 (3)	C4—C5—C6—C1	−0.3 (3)
C7—O1—C3—C4	−173.5 (2)	C4—C5—C6—C9	−176.89 (16)
C8—O2—C4—C3	165.72 (18)	C5—C6—C9—O4	−163.86 (15)
C8—O2—C4—C5	−13.3 (3)	C5—C6—C9—O3	17.3 (3)
C10—O4—C9—C6	10.7 (2)	C1—C6—C9—O4	19.5 (2)
C9—O4—C10—C13	−177.08 (14)	C1—C6—C9—O3	−159.38 (18)
C9—O4—C10—C11	−52.23 (19)	O4—C10—C11—C12	−171.59 (13)
C10—O4—C9—O3	−170.33 (15)	O4—C10—C13—S1A	50.92 (19)
C2—C1—C11—C10	144.44 (16)	C13—C10—C11—C1	−177.25 (14)
C2—C1—C6—C5	−1.3 (2)	O4—C10—C11—C1	63.07 (16)
C6—C1—C11—C12	−158.42 (15)	C11—C10—C13—C14A	109.1 (4)
C2—C1—C6—C9	175.27 (16)	C13—C10—C11—C12	−51.91 (19)
C11—C1—C2—C3	−179.11 (16)	O4—C10—C13—C14A	−131.1 (4)
C11—C1—C6—C5	179.76 (15)	C11—C10—C13—S1A	−68.96 (19)
C11—C1—C6—C9	−3.7 (2)	C1—C11—C12—O5	−123.00 (19)
C2—C1—C11—C12	22.7 (2)	C1—C11—C12—O6	58.6 (2)
C6—C1—C11—C10	−36.70 (19)	C10—C11—C12—O5	116.78 (19)
C6—C1—C2—C3	2.1 (3)	C10—C11—C12—O6	−61.66 (18)
C1—C2—C3—C4	−1.3 (3)	S1A—C13—C14A—C15	1.0 (6)
C1—C2—C3—O1	178.82 (17)	C10—C13—C14A—C15	−177.2 (3)
C2—C3—C4—C5	−0.3 (3)	C13—C14A—C15—C16	−0.7 (7)
C2—C3—C4—O2	−179.43 (16)	C14A—C15—C16—S1A	0.1 (5)
O1—C3—C4—O2	0.5 (2)		

Symmetry codes: (i) *x*, *y*, *z*+1; (ii) −*x*, −*y*+2, −*z*+1; (iii) *x*, *y*−1, *z*; (iv) −*x*, −*y*+1, −*z*+1; (v) −*x*+1, −*y*+2, −*z*+1; (vi) *x*, *y*+1, *z*; (vii) −*x*+1, −*y*+1, −*z*; (viii) −*x*+1, −*y*+1, −*z*+1; (ix) *x*, *y*, *z*−1; (x) *x*+1, *y*, *z*−1; (xi) *x*−1, *y*, *z*+1.

### Hydrogen-bond geometry (Å, °)

**Table:**

*D*—H···*A*	*D*—H	H···*A*	*D*···*A*	*D*—H···*A*
O6—H6···O3^vi^	0.86 (3)	1.84 (2)	2.658 (2)	159 (3)
C2—H2···O5^v^	0.93	2.54	3.465 (2)	172
C10—H10···O6^ii^	0.98	2.54	3.475 (2)	159
C11—H11···Cg4^viii^	0.98	2.61	3.525 (2)	156

Symmetry codes: (vi) *x*, *y*+1, *z*; (v) −*x*+1, −*y*+2, −*z*+1; (ii) −*x*, −*y*+2, −*z*+1; (viii) −*x*+1, −*y*+1, −*z*+1.

## References

[bb1] Altomare, A., Burla, M. C., Camalli, M., Cascarano, G. L., Giacovazzo, C., Guagliardi, A., Moliterni, A. G. G., Polidori, G. & Spagna, R. (1999). *J. Appl. Cryst.* **32**, 115–119.

[bb2] Bogdanov, M. G., Gocheva, B. T., Dimitrova, D. B. & Palamreva, M. D. (2007*a*). *J. Heterocycl. Chem.* **44**, 673–677.

[bb3] Bogdanov, M. G., Kandinska, M. I., Dimitrova, D. B., Gocheva, B. T. & Palamareva, M. D. (2007*b*). *Z. Naturforsch. Teil C*, **62**, 477–482. 10.1515/znc-2007-7-804 17913060

[bb4] Bogdanov, M. G. & Palamareva, M. D. (2004). *Tetrahedron*, **60**, 2525–2530.

[bb5] Cremer, D. & Pople, J. A. (1975). *J. Am. Chem. Soc.* **97**, 1354–1358.

[bb6] Farrugia, L. J. (1997). *J. Appl. Cryst.* **30**, 565.

[bb7] Farrugia, L. J. (1999). *J. Appl. Cryst.* **32**, 837–838.

[bb8] Sheldrick, G. M. (2008). *Acta Cryst.* A**64**, 112–122. 10.1107/S0108767307043930 18156677

[bb9] Stoe & Cie (2002). *X-AREA* and *X-RED32* Stoe & Cie, Darmstadt, Germany.

